# The Frog Skin-Derived Antimicrobial Peptide Esculentin-1a(1-21)NH_2_ Promotes the Migration of Human HaCaT Keratinocytes in an EGF Receptor-Dependent Manner: A Novel Promoter of Human Skin Wound Healing?

**DOI:** 10.1371/journal.pone.0128663

**Published:** 2015-06-12

**Authors:** Antonio Di Grazia, Floriana Cappiello, Akiko Imanishi, Arianna Mastrofrancesco, Mauro Picardo, Ralf Paus, Maria Luisa Mangoni

**Affiliations:** 1 Istituto Pasteur-Fondazione Cenci Bolognetti, Department of Biochemical Sciences, Sapienza University of Rome, Rome, Italy; 2 Centre for Dermatology Research, Institute of Inflammation and Repair, University of Manchester, Manchester, United Kingdom; 3 Laboratory of Cutaneous Physiopathology and Integrated Center of Metabolomics Research, San Gallicano Dermatologic Institute, Rome, Italy; 4 Department of Dermatology, University of Münster, Münster, Germany; University of Kiel, GERMANY

## Abstract

One of the many functions of skin is to protect the organism against a wide range of pathogens. Antimicrobial peptides (AMPs) produced by the skin epithelium provide an effective chemical shield against microbial pathogens. However, whereas antibacterial/antifungal activities of AMPs have been extensively characterized, much less is known regarding their wound healing-modulatory properties. By using an *in vitro* re-epithelialisation assay employing special cell-culture inserts, we detected that a derivative of the frog-skin AMP esculentin-1a, named esculentin-1a(1-21)NH_2_, significantly stimulates migration of immortalized human keratinocytes (HaCaT cells) over a wide range of peptide concentrations (0.025–4 μM), and this notably more efficiently than human cathelicidin (LL-37). This activity is preserved in primary human epidermal keratinocytes. By using appropriate inhibitors and an enzyme-linked immunosorbent assay we found that the peptide-induced cell migration involves activation of the epidermal growth factor receptor and STAT3 protein. These results suggest that esculentin-1a(1-21)NH_2_ now deserves to be tested in standard wound healing assays as a novel candidate promoter of skin re-epithelialisation. The established ability of esculentin-1a(1-21)NH_2_ to kill microbes without harming mammalian cells, namely its high anti-*Pseudomonal* activity, makes this AMP a particularly attractive candidate wound healing promoter, especially in the management of chronic, often *Pseudomonas*-infected, skin ulcers.

## Introduction

Gene-encoded antimicrobial peptides (AMPs) are produced by all living unicellular and multicellular organisms; they serve key roles in innate immune defences and possess a wide spectrum of activities against bacteria, fungi and viruses [1–3]. Despite their enormous structural diversity, most AMPs share a net positive charge at neutral pH, a high content of hydrophobic residues and an amphipathic character [4,5]. In animals, they are predominantly expressed in the skin and the mucosal surfaces (e.g. the mouth, the eyes, the genito-urinary tract and the gut) where they form a chemical barrier between host tissues and the environment [6–8]. In addition, they are also produced by circulating immune cells such as leukocytes [9,10].

The skin is the environmentally most exposed organ and provides first-line defence against penetrating infectious microorganisms. In mammals, including humans, epidermal keratinocytes produce AMPs (e.g., the cathelicidin LL-37 and the beta-defensins hBD2 and hBD3) which are stored along with lipids within secretory granules called lamellar bodies [11–17]. Following skin wounding, infection and inflammation, keratinocyte lamellar bodies release their content of hydrophobic products and AMPs into intercellular spaces forming a chemical barrier against water loss and microbial attack [18–20]. Furthermore, infiltrating immune cells such as neutrophils and natural killer cells also contribute to the pool of AMPs in the skin [21–23].

Apart from their direct antimicrobial activity, mammalian AMPs display additional protective functions which have led to their classification as "host-defence peptides" [24]. These functions include the capability to stimulate the host immune system while suppressing the inflammatory response, as well as the ability to promote tissue repair through stimulation of epithelial cell migration [25–28].

In this respect, frog skin-derived peptides, including AMPs, may be of particular interest [29]. As a stratified squamous epithelium, the epidermis of adult frogs is histologically quite similar to that of humans [30]. Amphibian skin has a tissue repair and a defence system that enables wound healing without scarring [31] and contains a huge arsenal of pharmacological agents, including multiple neuropeptides and AMPs [32]. The AMPs are mainly stored within granules of the dermal glands controlled by the sympathetic nervous system and are secreted in response to stress or physical injury by a holocrine-type mechanism [33–35]. All frog species synthesize a unique set of AMPs, constituting families of 2–100 closely related members [36,37]. Over the past two decades, *in vitro* and *in vivo* experiments have demonstrated that frog-skin AMPs play a crucial role in maintaining the equilibrium of the natural microbial flora and that their synthesis is induced by microorganisms [38–40].

Recently, we have focused on the short variant of the frog-skin AMP esculentin-1a, esculentin-1a(1-21)NH_2_ [Esc(1-21), GIFSKLAGKKIKNLLISGLKG-NH_2_]. This consists of the first 20 amino acids of esculentin-1a (isolated from the skin of *Pelophylax lessonae/ridibundus*, formerly known as *Rana esculenta*) plus a glycinamide residue at its C-terminus [34,41–43]. This peptide possesses a wide spectrum of antimicrobial activity with demonstrated efficacy against both planktonic and biofilm forms of the Gram-negative bacterium *Pseudomonas aeruginosa*. *P*. *aeruginosa* is clinically important as a major pathogenic microorganism that causes various types of infections, such as those associated with the lungs, ocular surface, middle ear and skin wounds [44,45]. Mode of action studies have shown that membrane-perturbing activity is the major mechanism responsible for the killing action of Esc(1-21) on both phenotypes of this pathogen, thus limiting the induction of microbial resistance [46]. Resistance to cationic peptides whose mechanism of action is based on non-specific interaction with the anionic phospholipids of the bacterial membrane is considered difficult since it would involve drastic changes of the membrane lipid composition and potentially compromising the pathogen’s survival [46]. Note that compared to the extensively studied human skin AMP, LL-37 [47–50], the frog-skin AMP derived Esc(1-21) peptide may be clinically more attractive, for example due to the ability to preserve antimicrobial activity in biological fluids [51], and to its documented fast killing activity against a major human pathogen associated with chronic skin ulcers, *P*. *aeruginosa* [46,52]. However, it is as yet unknown whether Esc(1-21) has any wound healing-promoting properties.

As a first screening step towards exploring whether Esc(1-21) peptide is able to promote re-epithelialisation in the human system, we studied its ability to stimulate, *in vitro*, the migratory activity of human immortalized and primary epidermal keratinocytes in a modified scratch assay, and compared the results with those of LL-37. In order to establish whether a stereospecific mechanism based on the interaction with a chiral target was involved, the all-D-enantiomer of Esc(1-21) (containing all amino acid residues in the D configuration) was used. Furthermore, as already found for LL-37 [53], the involvement of the epidermal growth factor receptor (EGFR) and STAT3 protein in the peptide-induced signaling pathway controlling migration of HaCaT cells was also investigated.

## Materials and Methods

### Materials

Hoechst 33258, 3(4,5-dimethylthiazol-2yl)2,5-diphenyltetrazolium bromide (MTT), AG1478, mitomycin C; phalloidin-fluorescein isothiocyanate, LL-37 were from Sigma-Aldrich (St. Luis, MO); GM6001 was from Calbiochem (Merk Millipore, Germany).

### Frog skin peptides

Synthetic all-L and all-D Esc(1-21) peptides as well as the rhodamine-labeled all-L Esc(1-21) were purchased from Selleck Chemicals (Houston, TX, USA). Briefly, each peptide was assembled by step-wise solid-phase synthesis using a standard F-moc strategy and purified by RP-HPLC on a semipreparative C18-bonded silica column (Kromasyl, 5 μm, 100 Å, 25 cm × 4.6 mm) using a gradient of acetonitrile in 0.1% aqueous trifluoroacetic acid (from 25 to 100% in 30 min) at a flow rate of 1.0 ml/min. The product was obtained by lyophilization of the appropriate fraction. Analytical RP-HPLC indicated a purity >98%. The molecular mass was verified by using MALDI-TOF Voyager DE (Applied Biosystems, Carlsbad, CA, USA) as previously described [54].

### Cell culture

The extensively characterized human immortalized keratinocyte cell line, HaCaT (ATCC, USA) [55] was used in most assays. Cells were cultured in Dulbecco’s modified Eagle’s medium (DMEM) supplemented with 10% heat-inactivated fetal bovine serum (FBS), glutamine (4 mM) and 0.05 mg/ml gentamycin, at 37°C and 5% CO_2_, in 25-cm^2^ flasks.

Cultures of primary human epidermal keratinocytes (NHKs), derived from neonatal foreskins (at passage 4) were maintained in Medium 154 (Invitrogen, Life Sciences, Milan, Italy) supplemented with Human Keratinocyte Growth Supplement (HKGS, Invitrogen) plus antibiotics (100 μg/ml penicillin/streptomycin), 4 mM glutamine and Ca^2+^ (0.07 mM) in a humidified atmosphere containing 5% CO_2_ at 37°C, as previously described [56]. All treatments were performed in the same medium without HKGS to avoid any interference with the peptide's activity.

### Cell toxicity assay

The toxic effect of the investigated peptides on HaCaT cells was evaluated using the MTT colorimetric method [57]. MTT is a tetrazolium salt which is reduced to a formazan product by mitochondrial reductases giving a purple color. The intensity of the color is directly proportional to the number of metabolically-active cells. Keratinocytes were plated in triplicate wells of a microtiter plate, at 4 x 10^4^ cells/well in DMEM supplemented with 4 mM glutamine (DMEMg) and 2% FBS without antibiotic. After overnight incubation at 37°C and 5% CO_2_ atmosphere, the medium was replaced with 100 μl fresh serum-free DMEMg containing the peptides at different concentrations. The plate was incubated for 2h or 24h at 37°C and 5% CO_2_ atmosphere. Then, DMEMg was removed and replaced with Hank’s buffer (136 mM NaCl; 4.2 mM Na_2_HPO_4_; 4.4 mM KH_2_PO_4_; 5.4 mM KCl; 4.1 mM NaHCO_3_, pH 7.2, supplemented with 20 mM D-glucose) containing 0.5 mg/ml MTT. After 4 h incubation, the formazan crystals were dissolved by adding 100 μl of acidified isopropanol according to [57], and absorbance of each well was measured at 570 nm using a microplate reader (Infinite M200; Tecan, Salzburg, Austria).

### *In vitro* cell migration assay

The peptides' ability to stimulate migration of epithelial cells, *in vitro*, was studied according to a modified scratch assay, as described in [58–60]. Briefly, HaCaT cells (40,000) suspended in DMEMg supplemented with 10% FBS were seeded on each side of an ibidi culture insert for live cell analysis (Ibidi, Munich, Germany). Inserts were placed into wells of a 12-wells plate and incubated at 37°C and 5% CO_2_ to allow cells grow to confluence. Afterwards, inserts were removed with sterile tweezers to create a cell-free area (pseudo-"wound") of approximately 500 μm, whose re-epithelialisation by migrating HaCaT keratinocytes was quantitatively assessed; 1 ml serum-free DMEMg supplemented or not with the peptide at different concentrations was added. Cells were allowed to migrate in an appropriate incubator.

In the case of NHKs, 35,000 cells suspended in supplemented Medium 154 were seeded on each side of the ibidi culture insert. After overnight incubation at 37°C and 5% CO_2_ the medium was replaced with fresh medium without HKGS for 6 h. Afterwards, inserts were removed as described above and 1 ml of medium supplemented or not with the peptide at different concentrations was added. Primary keratinocytes were allowed to migrate in an appropriate incubator, as for HaCaT cells. All experiments were run three times in triplicates.

At different time intervals, fields of the pseudo-"wound" area were visualized under an inverted microscope (Olympus CKX41) at x 4 magnification and photographed with a Color View II digital camera. The percentage of cell-covered area at each time was determined by WIMASIS Image Analysis program. The migration speed was evaluated according to WIMASIS' instructions.

Pseudo-“wound” closure assays with HaCaT cells were also conducted by pre-treating these cells with 3 μM mitomycin C [60,61], 25 μM GM6001 [62,63] or 0.2 μM AG1478 inhibitor [53,64] in order to assess the contribution of cell proliferation, metalloproteinase activity or EGFR signaling in the peptide-induced migration of keratinocytes.

### Enzyme-linked immunosorbent assay (ELISA)

Phosphorylation of STAT-3 by all-L Esc(1-21) was analyzed by an ELISA assay according to the manufacturer's protocol (Phospho Tyr 705-Stat3 ELISA, RayBiotech, Norcross, GA, USA). Briefly, subconfluent HaCaT keratinocytes (about 1x 10^6^ cells in a 6-cm dish plate) were treated or not with 0.25 μM peptide in DMEMg for 20 min. Afterwards, cells were lysed with lysis buffer supplemented with proteases and phosphatases inhibitors. Samples were centrifuged at 12,600 g and the supernatants were added to wells of a microtiter plate coated with anti-STAT3 antibody. After 2.5 h incubation at room temperature, the supernatant was discarded, the wells were washed and biotinylated anti-STAT3 (Tyr 705) antibody was added to each well, for 1h at room temperature, to detect only phosphorylated STAT3 (Tyr 705). After washing away unbound antibody, horseradish peroxidase conjugated streptavidin was added to each well for 1h. The wells were washed again and a substrate (3,3’,5,5’-tetramethylbenzidine) solution was added. Color developed in proportion to the amount of bound phosphorylated STAT3 (Tyr 705). The reaction was stopped after 30 min incubation and the intensity of the color was measured with a microplate reader (Infinite M200; Tecan, Salzburg, Austria) at 450 nm.

### Fluorescence microscopy

HaCaT cells (40,000) were seeded on coverslips for 24h in DMEMg supplemented with 10% FBS, at 37°C and 5% CO_2_. After 24h, cells were washed with phosphate buffered saline (PBS) and treated with rhodamine-labeled Esc(1-21) (4 μM in serum-free DMEMg) at 37°C and 5% CO_2_. After different time intervals (30 min and 24h), samples were washed with PBS and fixed with 3.7% formaldehyde for 10 min at +4°C. Afterwards, they were washed with PBS and stained with 2 μg/ml Hoechst 33258 for 10 min at room temperature [65]. The coverslips were placed on a glass slide with buffered glycerol and visualized under a fluorescence microscope (Keyence Biozero-8000 Microscope; Keyence Corporation, Osaka, Japan). Hoechst and rhodamine-labeled Esc(1-21) were visualized using laser wavelength of 360 and 560 nm respectively. All images were taken using objective lens of 60X and zoom.

### Statistical analyses

Data were collected and pooled from three independent experiments. Quantitative data are expressed as the mean ± SEM. Statistical analysis was performed using Student's *t* test or two-way analysis of variance (ANOVA) with PRISM software (GraphPad, San Diego, CA). Differences were considered to be statistically significant for a p value < 0.05.

## Results

### Esc(1-21) enantiomers do not exhibit significant toxicity towards HaCaT keratinocytes

**Fig 1:**
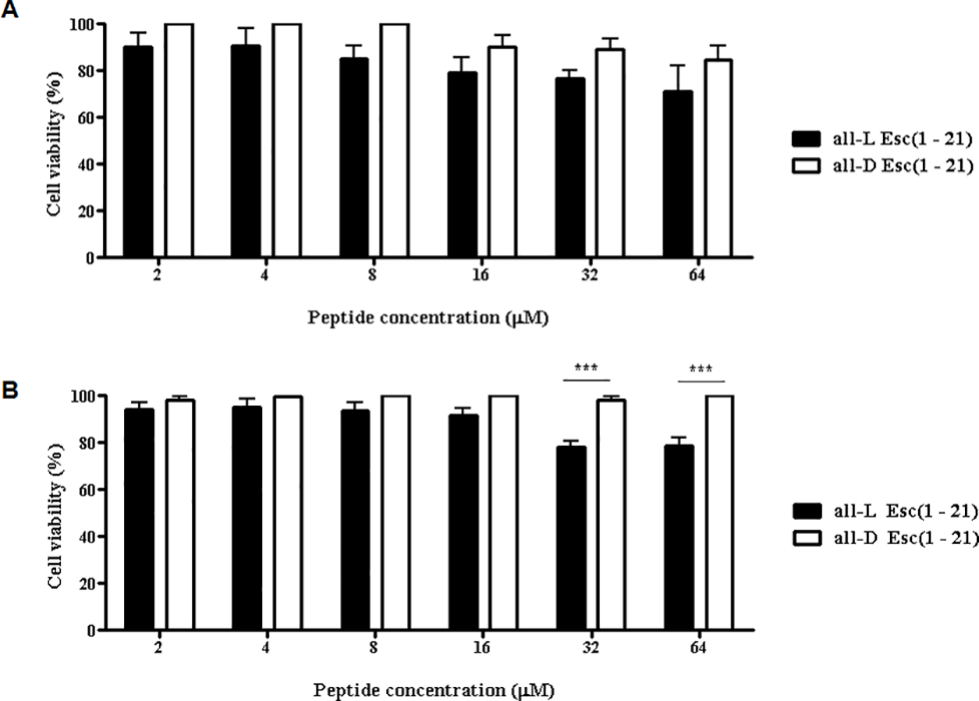
Effect of all-L and all-D Esc(1-21) peptides at different concentrations on the number of metabolically-active HaCaT cells after a short (A) or long (B) term treatment. Cells were plated in wells of a microtiter plate, at 4x10^4^ cells/well. After overnight incubation at 37°C in a 5% CO_2_ atmosphere, the medium was replaced with 100 μl fresh medium supplemented with the peptides at different concentrations. After 2h (A) or 24h (B) of peptide treatment, cell viability was determined by the MTT reduction to insoluble formazan (see Materials and Methods for additional information). Cell viability is expressed as percentage with respect to the vehicle-treated control cells. Data points represent the mean of triplicate samples ± SEM. The level of statistical significance between all-L and all-D peptides is indicated as follows: ***, p<0.001.

In order to assess, first, the toxicity of Esc(1-21) enantiomers, the number of metabolically-active HaCaT cells after a short (2h) and long (24h) treatment interval was studied. Neither peptide showed any marked reduction in the number of metabolically-active keratinocytes after a 2h incubation at concentrations of 2 to 64 μM, and the difference between the two was not statistically significant (Fig 1A). In contrast, after a 24h interval, only the all-L peptide showed slight toxicity at 32 μM and 64 μM (Fig 1B), causing approximately a 20% reduction in the percentage of metabolically-active cells compared to the all-D Esc(1-21) (p<0.001). Note that LL-37 led to complete inhibition of HaCaT cells metabolism at 200 μg/ml (~44 μM) [66]. This shows that only the all-L Esc(1-21) has limited toxicity towards HaCaT cells, but that this is significantly lower than that reported for LL-37 [66].

### All-L Esc(1-21), but not its all-D enantiomer, stimulates HaCaT cell migration

**Fig 2:**
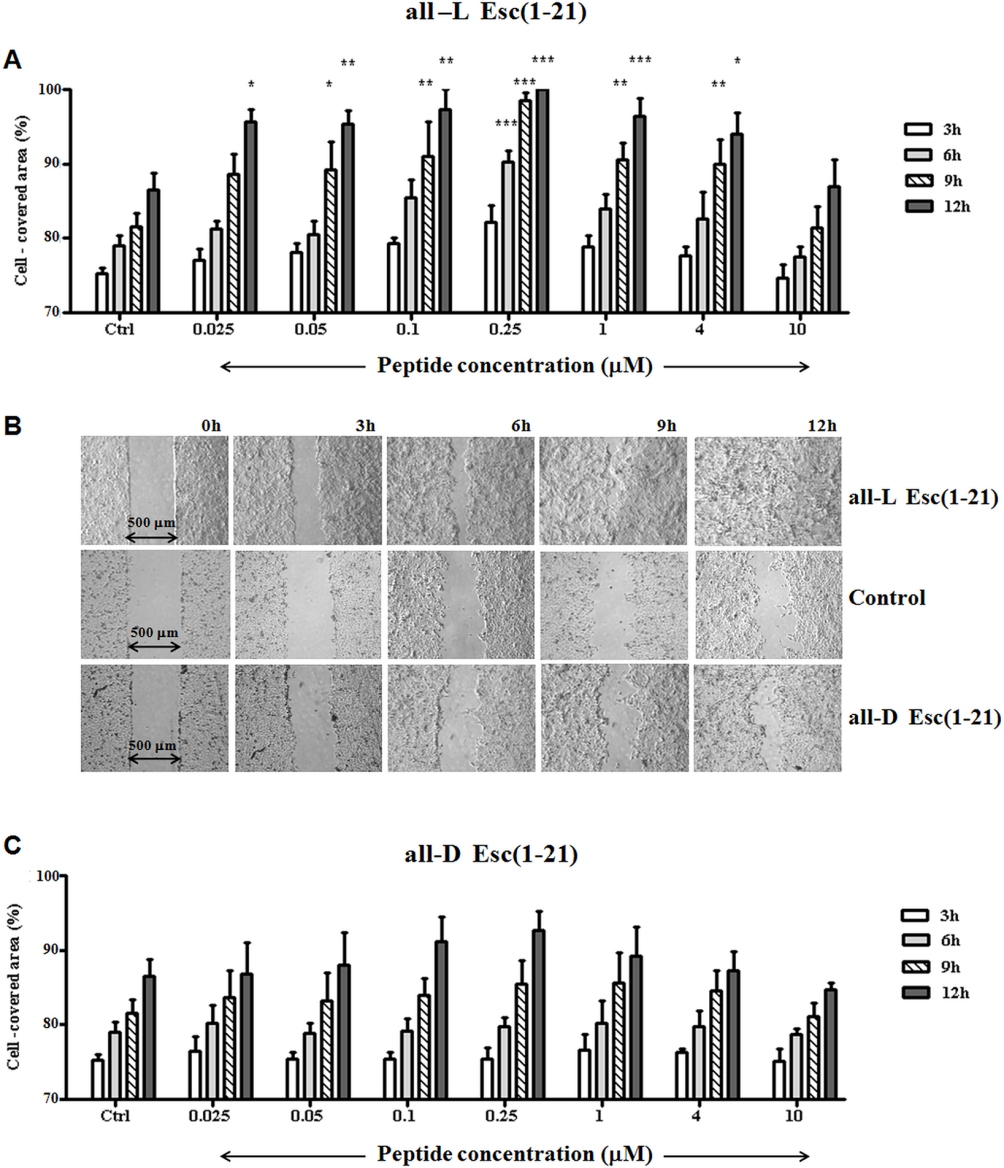
Effect of all-L and all-D Esc(1-21) on the closure of a pseudo-"wound" field produced in a monolayer of HaCaT cells. HaCaT cells were seeded in each side of an ibidi culture insert and grown to confluence. Afterwards, they were treated with all-L (A) or all-D (C) peptide at different concentrations, as indicated. Cells were photographed at the time of insert removal (0 h) and examined for cell migration after 3, 6, 9 and 12h from peptide addition. The percentage of cell-covered area at each time point is reported on the y-axis. Control (Ctrl) is given by vehicle-treated cells. All data are the mean of three independent experiments ± SEM. The levels of statistical significance between Ctrl and peptide-treated samples are indicated as follows: *, p<0.05; **, p<0.01;***, p<0.001. (B): micrographs showing representative results of pseudo-"wound" closure induced upon treatment of keratinocytes with 0.25 μM all-L Esc(1-21) or all-D Esc(1-21) with respect to the Ctrl sample.

To evaluate, next, the ability of different concentrations of both isomers to promote cell migration, an *in vitro* cell migration assay was performed by means of special plastic inserts and a modified scratch assay design [55] to create a 500 μm wide gap within a monolayer of HaCaT cells. The all-L peptide promoted complete coverage of the pseudo-"wound" field in about 9–12h with a bell-shaped dose-response curve (Fig 2A). The optimal concentration allowing gap closure was 0.25 μM (Fig 2A and 2B). In contrast, no statistically significant difference in the cell-covered area was measured between the all-D Esc(1-21)-treated samples and the vehicle (medium)-treated control group (Fig 2B and 2C). That the all-D enantiomer did not significantly promote re-epithelialisation of the pseudo-"wound" area *in vitro* suggests stereospecificity of the mechanism of all-L Esc(1-21)-induced keratinocyte migration.

### All-L Esc(1-21) also stimulates “wound” re-epithelialisation by primary human epidermal keratinocytes *in vitro*

**Fig 3:**
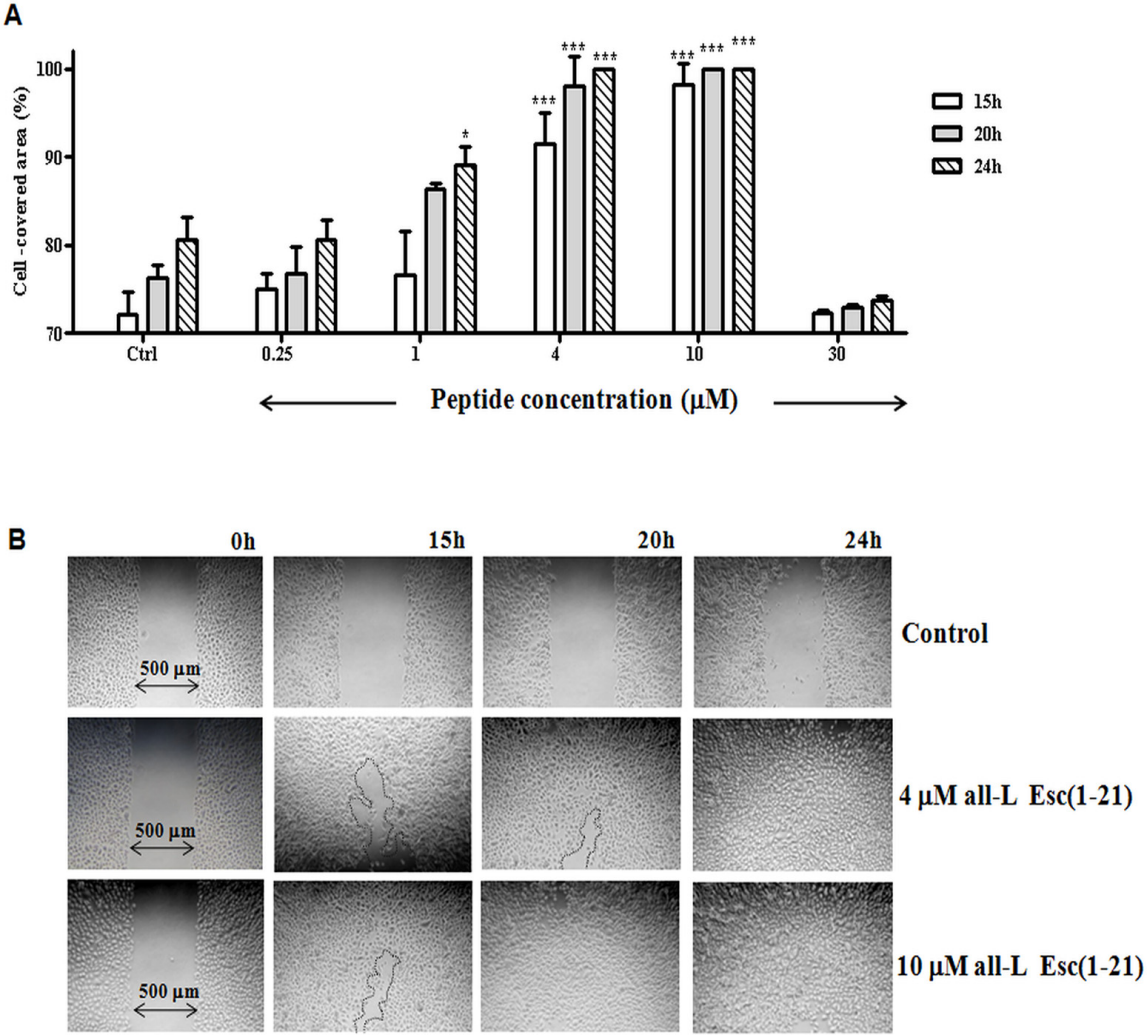
Effect of all-L Esc(1-21) on the closure of a pseudo-"wound" field produced in a monolayer of primary human keratinocytes. (A): two monolayers of primary keratinocytes separated by a defined distance were treated with the peptide at different concentrations, as indicated. Cells were photographed at the time of insert removal (0 h) and examined for cell migration after 15, 20 and 24h from peptide addition. The percentage of cell-covered area at each time point is reported on the y-axis. Control (Ctrl) is given by vehicle-treated cells. All data are the mean of three independent experiments ± SEM. The levels of statistical significance between Ctrl and peptide-treated samples are indicated as follows: *, p<0.5; ***, p<0.001. (B): micrographs showing representative results upon treatment of primary keratinocytes with all-L Esc(1-21) (4 μM and 10 μM) with respect to the Ctrl sample. The black line marks off the cell-free area in the peptide-treated samples after 15 and 20 h. No cell-free area was noted in samples treated with 10 μM peptide after 20 and 24h.

As the biological properties of cell lines differ from those of primary cells, the pseudo-"wound" healing activity of all-L Esc(1-21) was also tested on monolayers of primary human epidermal keratinocytes. Importantly, the peptide was found to promote re-epithelialisation of the "wound" gap area within 24h at a concentration range between 4 μM and 10 μM (Fig 3A). The most effective peptide dosage (10 μM) completely restored integrity of the NHK monolayer within 20 h (Fig 3B).

### LL-37 stimulates HaCaT cell migration at a narrower concentration range and less efficiently than all-L Esc(1-21)

**Fig 4:**
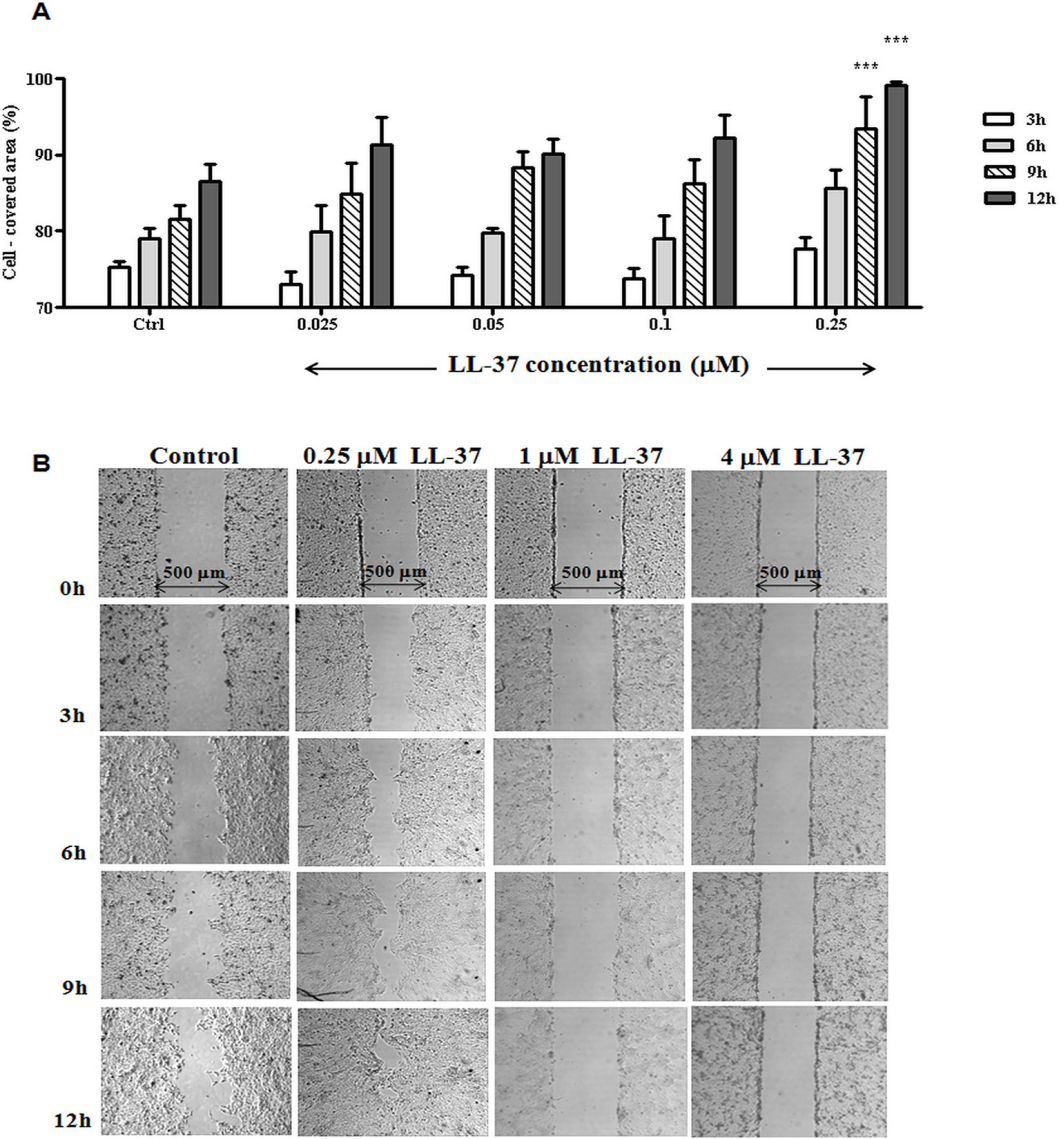
Effect of LL-37 on the closure of a pseudo-"wound" field produced in a monolayer of HaCaT cells. (A): two monolayers of HaCaT cells separated by a defined distance were treated with LL-37 at different concentrations, as indicated. Cells were photographed at the time of insert removal (0 h) and examined for cell migration after 3, 6, 9 and 12h from peptide addition. The percentage of cell-covered area at each time point is reported on the y-axis. Control (Ctrl) is given by vehicle-treated cells. All data are the mean of three independent experiments ± SEM. The level of statistical significance between Ctrl and peptide-treated samples is indicated as follows: ***, p<0.001. (B): micrographs showing representative results upon treatment of keratinocytes with LL-37 (0.25 μ M, 1 μ M and 4 μ M) with respect to the Ctrl sample.

In order to compare all-L Esc(1-21) with the most extensively investigated human skin AMP, the cells' migratory activity caused by LL-37 was subsequently analyzed (Fig 4A). LL-37 is a well-known human AMP with a demonstrated wound healing effect [67] and ability to promote the migration of several different mammalian cell types, including keratinocytes [68] and corneal epithelial cells [69]. However, LL-37 activities have not previously been evaluated under the present experimental conditions, i.e. using the culture insert scratch assay modification employed here [55].

In agreement with a published report [53], LL-37 was able to stimulate the closure of the pseudo-"wound" field within 12h at an optimal concentration of 0.25 μM (Fig 4A and 4B). Importantly, however, the percentage of the re-epithelialized area calculated at different time intervals following peptide addition was higher for all-L Esc(1-21) (Fig 2A), with a front speed migration equal to 20 μm/h, compared to 17 μm/h estimated for LL-37. No statistically significant difference was obtained between vehicle-treated samples and those incubated with LL-37 at concentrations lower than 0.25 μM (Fig 4A). Furthermore, the cell migration activity was completely inhibited by LL-37 at concentrations of 1 μM and above (Fig 4B). Therefore, under the current assay conditions, LL-37 is less efficient than all-L Esc(1-21) in promoting migration of HaCaT cells *in vitro* and exhibits a narrower “therapeutic” window.

### All-L Esc(1-21) primarily promotes keratinocyte migration *in vitro*

**Fig 5:**
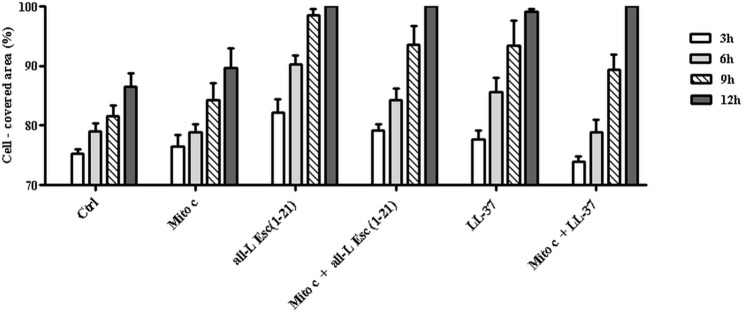
Effect of mitomycin C (Mito) on the peptides-mediated closure of a pseudo-"wound" field produced in a HaCaT cells monolayer. (A) After removal of the ibidi culture insert, HaCaT cell monolayers were pre-incubated or not with 3 μM Mito for 30 min and subsequently treated with 0.25 μM all-L Esc(1-21) or LL-37. Cells incubated with medium served as control (Ctrl). Samples were photographed at different time intervals, as indicated in the legends to Figs 2 and 4, and the percentage of cell-covered area was calculated and reported on the y-axis. All data are the mean of three independent experiments ± SEM. No statistical significance was found between samples treated with the peptide alone or pre-incubated with Mito.

Next, we examined potential mechanisms that may underlie the re-epithelialisation-promoting properties of all-L Esc(1-21). In order to abrogate cell proliferation, HaCaT cells were pre-treated with the cell proliferation blocker, mitomycin C. As shown in Fig 5, this did not significantly delay re-epithelialisation of the pseudo-“wound” area in monolayer culture produced by either 0.25 μM all-L Esc(1-21) or LL-37, within 12h. When the activity of mitomycin C was checked independently, it was noted that this treatment switched the morphology of HaCaT cells to a round cell shape, a recognized stop signal for keratinocyte cell division [70], without influencing their viability (data not shown).

**Fig 6:**
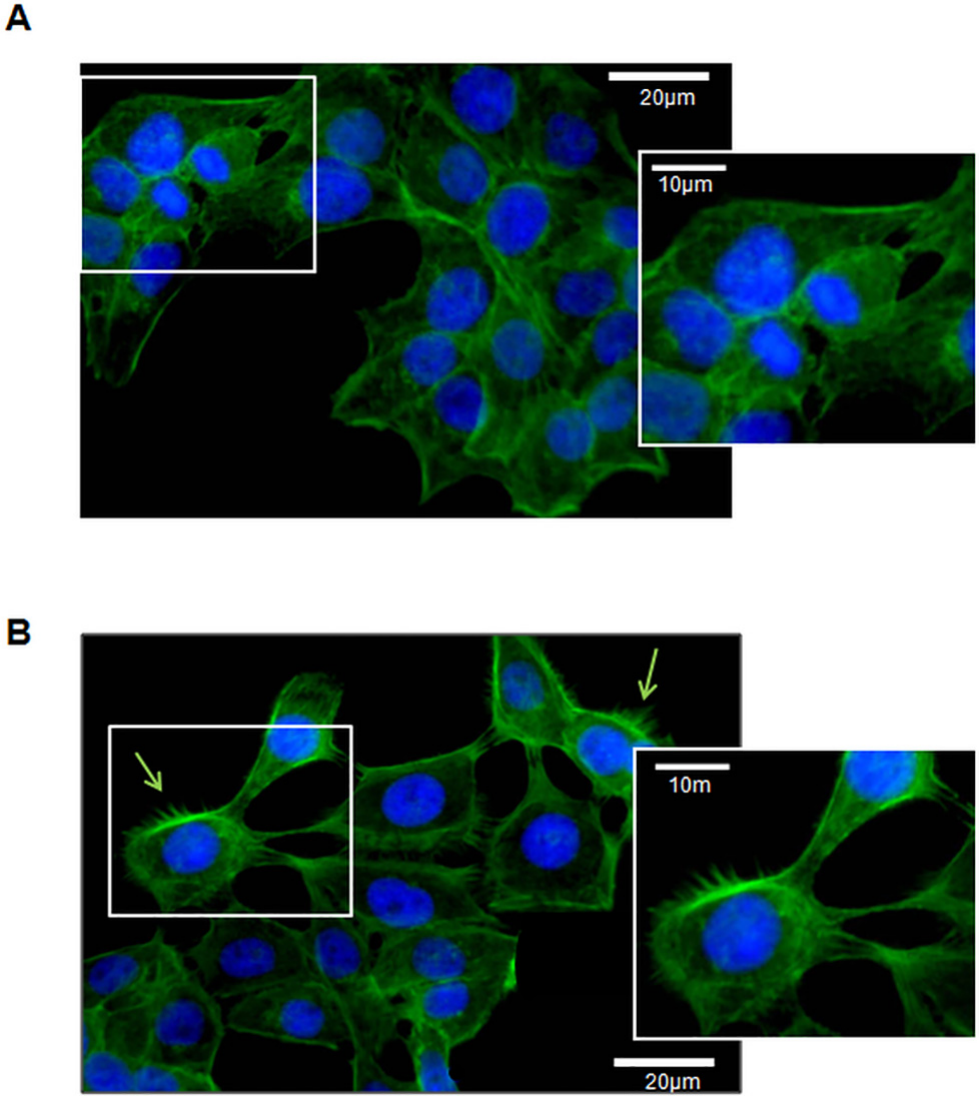
Effect of all-L Esc(1-21) on the phenotype of HaCaT cells. Cells (150,000) were seeded on a glass coverslip and treated or not with 0.25 μM peptide in DMEMg for 12h at 37°C and 5% CO_2_. Afterwards, samples were washed three times with PBS and fixed with 3.7% formaldehyde for 10 min at +4°C. Then, they were permeabilized with 0.1% Triton X-100 for 10 min at room temperature and stained with phalloidin-fluorescein isothiocyanate (40 μM in PBS) for 30 min at room temperature, to visualize the cytoskeleton. The nuclei were stained by adding 50 μl of Hoechst 33258 (2 μg/ml) for 10 min at room temperature. The coverslips were mounted on slides and observed under a fluorescent microscope (KOZO OPTICS XJF800) at 40 x magnification and photographed with a Color View II digital camera. (A): vehicle-treated control cells; (B) peptide-treated cells. Insets represent the magnification of the image portion indicated by the white frame. rows indicate filopodial formation.

Furthermore, compared to vehicle-treated HaCaT cells (Fig 6A), typical morphological changes associated with a migratory phenotype [71,72] were detected in HaCaT cells upon treatment with all-L Esc(1-21). Notably, these became visible through the formation of cytoplasmic protrusions developing into filopodial extensions (Fig 6B), quite similar to the phenotypic effects that have been reported for LL-37 [68]. Overall, these observations suggest that the re-epithelialisation-promoting properties of all-L Esc(1-21) primarily result from its ability to stimulate keratinocyte migration, rather than keratinocyte proliferation.

### All-L Esc(1-21) stimulates migration of HaCaT keratinocytes in a EGFR-dependent manner

**Fig 7:**
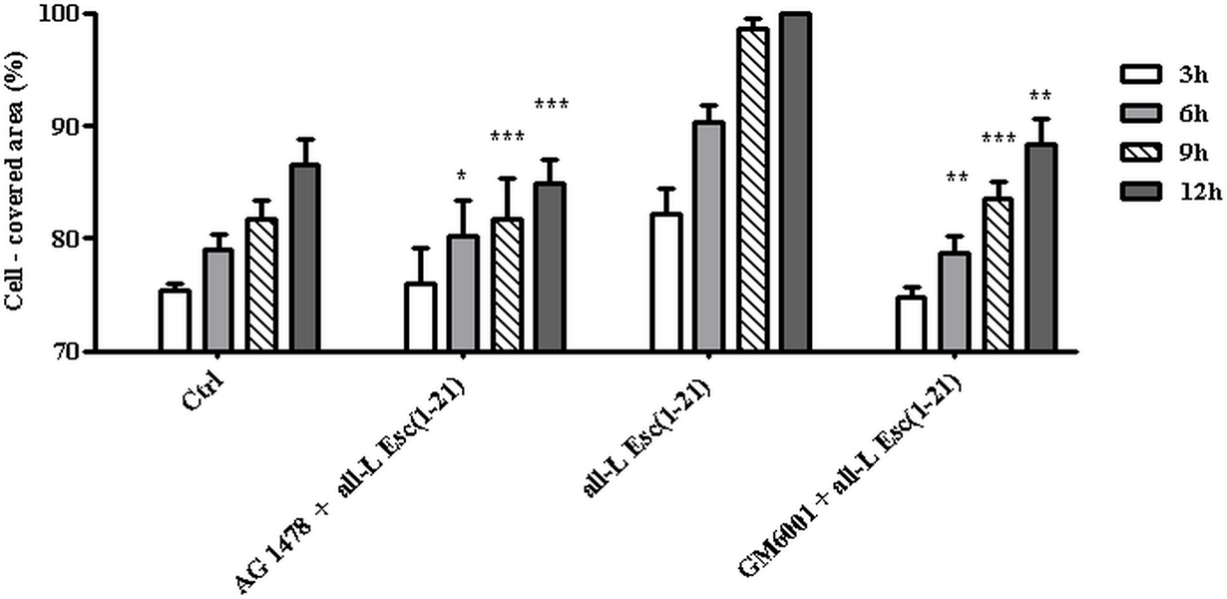
Effect of AG1478 and GM6001 inhibitors on the all-L Esc(1-21)-mediated closure of a pseudo-"wound" field produced in a HaCaT cells monolayer. After removal of the ibidi culture insert, HaCaT cell monolayers were pre-incubated with 0.2 μM AG1478 for 15 min or with 25 μM GM6001 for 30 min and subsequently treated with 0.25 μM all-L Esc(1-21). Cells incubated with medium served as control (Ctrl). Samples were photographed at different time intervals, as indicated in the legend to Fig 2, and the percentage of cell-covered area was calculated and reported on the y-axis. All data are the mean of three independent experiments ± SEM. The levels of statistical significance between inhibitor-pretreated groups and samples treated with the peptide alone are indicated as follows:*, p<0.05; **, p< 0.01; ***, p< 0.001.

To explore whether EGFR played a similar role in the signaling transduction cascade controlling the Esc(1-21)-stimulated migration of cells as it had been reported for LL-37 [53], HaCaT cells were pre-incubated with the tyrosine kinase inhibitor of EGFR, AG1478 [64], and were subsequently exposed to the all-L enantiomer. As illustrated in Fig 7, the peptide-promoted migration of HaCaT cells was significantly impaired by inhibiting EGFR-mediated signaling, suggesting that Esc(1-21)-stimulated re-epithelialisation requires EGFR activation.

Since metalloproteinases have been shown to cleave membrane-anchored EGFR ligands and to be involved in EGFR trans-activation [73], the effect of the metalloproteinase inhibitor GM6001 [62,63] was evaluated on the peptide-induced keratinocyte migration. Pretreatment of HaCaT cells with GM6001 indeed counteracted the Esc(1-21)-stimulated re-epithelialisation of the pseudo-"wound" area in keratinocyte monolayer (Fig 7) indicating a contribution of metalloproteinase activity in the peptide-induced stimulation of keratinocytes migration.

Hereafter, we determined whether the intracellular pathway involving the activation of STAT3 protein that has previously been demonstrated for LL-37 [53], is also implicated in Esc(1-21)-stimulated re-epithelialisation. To this end, an ELISA assay was performed to assess the level of STAT3 phosphorylation at Tyr 705 (active form) [74], after a 20 min incubation of HaCaT cells with the maximally effective concentration of all-L Esc(1-21) (0.25 μM). Keratinocytes treated with 0.25 μM LL-37 were included as a positive control.

**Fig 8:**
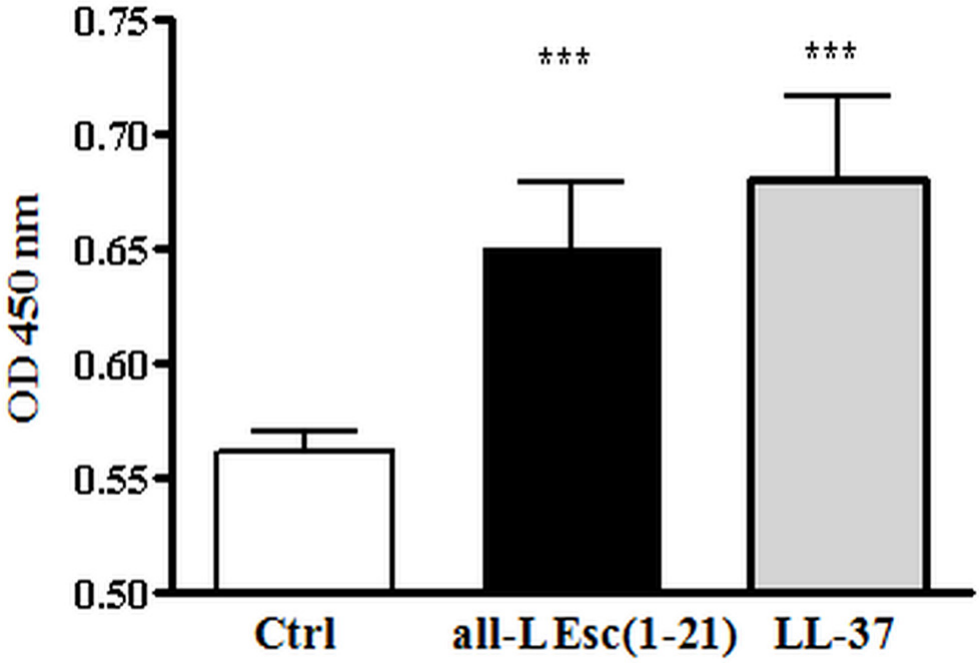
Phosphorylation of STAT3 by all-L Esc(1-21). About 1 x 10^6^ keratinocytes were stimulated with 0.25 μM all-L Esc(1-21) or LL-37 for 20 min. Control (Ctrl) was given by vehicle-treated cells. Cells were harvested into lysis buffer and phospho-STAT3 (Tyr 705) was evaluated by performing an ELISA assay (see Materials and methods). All data are the mean of three independent experiments ± SEM. The level of statistical significance between peptide-treated samples and Ctrl is indicated as follows: ***, p < 0.001.

This showed that the amount of phospho-STAT3(Tyr 705) was significantly higher in Esc(1-21)-treated cells compared to vehicle-treated controls (p< 0.001) and similar to that induced by LL-37 (Fig 8). This indicates that Esc(1-21)-promoted HaCaT cell migration, again similar to LL-37 [53], also involves STAT3 phosphorylation.

### All-L Esc(1-21) efficiently penetrates into the cytoplasm of HaCaT cells

**Fig 9:**
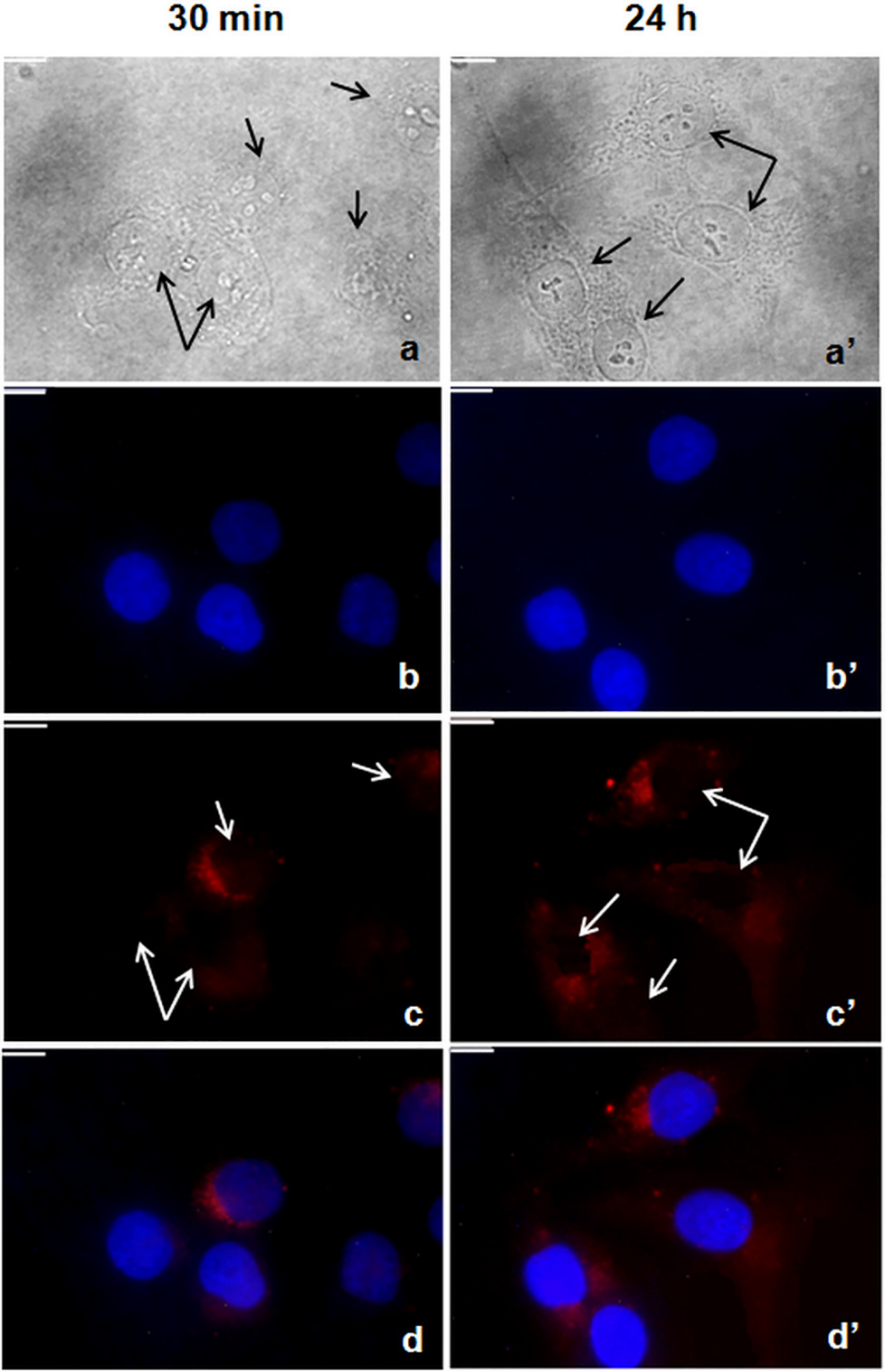
Images of HaCaT cells treated with rhodamine-labeled all-L Esc(1-21) at different times (30 min and 24h). Panels A and A' show bright field images. Black arrows indicate keratinocytes. B and B' show the Hoechst fluorescence signal which indicates the nuclei position. Panels C and C' show the distribution of the rhodamine-labeled Esc(1-21). Red fluorescence is not detectable at the level of nuclei (white arrows), but rather in the cytoplasm and mainly concentrated at the nuclei periphery, already after a short incubation time (C). Panels D and D' show the overlay of the two fluorescent probes. All images are z section images taken from the mid-cell height. All bars indicate 10 μm.

As this may affect the biological activities of this AMP *in vivo*, we finally investigated whether Esc(1-21) remains localized on the surface of cultured keratinocytes or is internalized into the cytoplasm or nucleus. HaCaT cells were exposed to rhodamine-labeled Esc(1-21) and stained with Hoechst 33258 dye so as to permit the differential visualization of the AMP and cell nuclei, respectively, by fluorescence microscopy. This revealed that the fluorescently labelled peptide was distributed inside the HaCaT cells and concentrated mainly at the nuclear periphery within 30 min after administration to the culture medium (Fig 9 left panels), without entering into the nucleus even 24h after its addition to the cells (Fig 9 right panels). Similar results were obtained with another class of amphibian skin AMPs, i.e. the temporins [55], suggesting that after membrane binding and indirect activation of EGFR, Esc(1-21) and likely other frog skin linear peptides are internalized into the keratinocyte cytoplasm.

## Discussion

Here, we provide the first evidence that a peptide fragment derived from the frog-skin AMP esculentin-1a, esculentin-1a(1-21)NH_2,_ promotes the re-epithelialisation of surrogate scratch “wounds” by human keratinocytes *in vitro*, notably both in HaCaT cells and in primary epidermal keratinocytes, and this more effectively and at much lower cell toxicity than the prototypic human skin AMP, LL-37. Importantly, migration of HaCaT cells upon addition of Esc(1-21) occurs over a wider peptide concentration range (from 0.025 to 4 μM) compared to LL-37 which completely loses activity at 1 and 4 μM (Fig 4). Furthermore, although 0.25 μM is the optimal concentration for both AMPs, the cell migration rate induced by Esc(1-21) is higher than that of LL-37.

This introduces Esc(1-21) as a new candidate wound healing promoter. Our study is in general agreement with earlier reports that other frog skin-derived peptides can promote epithelial cells migration [29,55] and wound healing in murine models, namely through the release of transforming growth factor β1 [75]. However, the current study is the first to show that an esculentin-1a-derived peptide promotes keratinocyte migration via an EGFR-dependent signaling pathway.

Mechanistically, we show that the re-epithelialisation promoted by Esc(1-21) is stereospecific, possibly based on a stereoselective interaction with chiral targets, such as proteins [25,76]. Importantly, cell proliferation does not significantly contribute to the re-epithelialisation-promoting effect of Esc(1-21), which appears to be mainly dependent on the stimulation of keratinocyte migration. As previously shown for LL-37 [53], the promotion of keratinocyte migration by Esc(1-21) requires activation of an EGFR signaling pathway. Note that erbB4 receptor (another member of the EGFR family), whose activation can be hampered by the EGFR inhibitor employed here (AG1478) [77–79], is not expressed in human keratinocytes and HaCaT cells [80,81]. Our *in vitro* assays have also indicated a contribution of metalloproteinase activity in the Esc(1-21)-induced migration of keratinocytes. This raises the question whether the re-epithelialisation-promoting activities of this AMP require cleavage of membrane-bound EGFR ligands and/or receptor trans-activation by metalloproteinases.

Furthermore, one of the signaling events implicates tyrosine phosphorylation and activation of STAT3 protein [82]. This is in line with the previous demonstration that EGFR-induced cell migration is mediated predominantly by the STAT-pathway in keratinocytes and that STAT3 plays an essential role for skin remodeling and wound healing [82,83].

Yet, multiple additional molecular mechanisms might participate in the enhanced migration of keratinocytes after exposure to Esc(1-21), such as the PI3K/Akt signaling pathway, likely mediated not only through activation of EGFR but also through the induction of G-protein-coupled receptor FPRL-1 [68]. Therefore, additional experiments are needed to clarify the exact intracellular signaling events that govern Esc(1-21)-promoted keratinocyte migration. In this context, it is interesting to note that our data also show that Esc(1-21) quickly translocates into the cytoplasm via yet to be discovered mechanisms, possibly upon interaction with the cell membrane and activation of EGFR; this may trigger further signaling pathways controlling keratinocyte migration and efficient re-epithelialisation. However, it can currently not be excluded that this peptide is also capable of integrating into the cell membrane (without causing cytotoxicity) and to subsequently enter into the cytosol, or that it can be internalized via endocytosis.

The current work also encourages one to re-evaluate the potential overall benefits of frog skin-derived AMPs in clinical medicine [28]. Skin secretions from many species of Anura (frogs and toads), especially those belonging to the Hylidae and Ranidae family, are among the most abundant sources for biologically-active peptides, including AMPs, presently numbering more than a thousand [84]. Esc(1-21) in particular has been recently identified as a derivative of the longer peptide esculentin-1a, with clinically attractive features for a potential new therapeutic agent. Remarkably, Esc(1-21) has the capability to preserve antibacterial activity even at high salt concentrations [46] as well as in the presence of serum and tears [51]. This is in sharp contrast to the properties of the key human AMPs, hBD-2 and LL-37, which completely lose their antimicrobial efficacy at the high ionic strength (e.g., 100 mM monovalent cations) found at many body sites [85], such as the sputum, airway surfaces and serum/plasma [86–90]. Furthermore, their antimicrobial activity is drastically reduced in biological fluids [91].

Although these mammalian AMPs certainly function as powerful microbicidal agents at the high concentration (mg/ml) present in the phagolysosomes of neutrophils, they probably act as immunomodulators at those physiological setting e.g. inflammatory sites, generally containing high levels of mono and bivalent cations, where they are released by degranulation at lower concentrations [47,90]. This would explain the lack of antibacterial activity of these AMPs when tested, *in vitro*, under conditions (i.e. peptide concentrations and culture media) that better mimic those encountered in their natural environment [88,91–94]. In contrast, the estimated concentration of peptides in the skin mucus of resting (non-stressed) frogs is within the concentration range that shows antimicrobial activity in *vitro* [95], supporting the notion that AMPs produced in amphibian skin provide remarkable chemical weapons against skin pathogens [96]. This may be especially important for newly metamorphosed frogs in which the adaptive immune system is still immature [35,97]. Another advantageous feature of Esc(1-21) compared to classical mammalian AMPs is that, given its short sequence, it may be possible to produce this AMP more economically. Finally, we should recall that Esc(1-21) is significantly less cytotoxic than LL-37 toward HaCaT cells, in agreement with literature data showing that human cathelicidins become harmful to mammalian cells, when used at high concentrations [98].

Given the complexity of the recognized individual stages of skin wound healing *in vivo* [99], it is evident that even the re-epithelialisation component of skin wound healing alone can be very incompletely reproduced *in vitro*, i.e. in the absence of fibroblasts, immunocytes, and functional perfusion and innervation. Therefore, the current results need to be followed-up, next, at the preclinical research level in more complex human skin assays, using for example the punch-in-a-punch design of organ-cultured full-thickness human skin [29]. However, the ibidi culture insert system used here [55] provides an objective and highly reproducible initial experimental screening tool for the quantitative evaluation of human keratinocyte migration and its manipulation by candidate wound healing promoters. This simplified *in vitro*-surrogate assay for re-epithelialisation may be preferable over the classical scratch assay method, where the gap width created with a plastic pipette tip is highly dependent on the pressure applied and where detached cells can easily form clumps at the edges of the scratch, affecting the rate of cell migration, thus giving potentially confounding results.

In summary, here we provide the first evidence that Esc(1-21) significantly and more effectively than LL-37 stimulates human keratinocyte migration in an *in vitro* assay, likely in manner that involves activation of EGFR and STAT3 protein. The established ability of Esc(1-21) to kill microbes without harming mammalian cells, namely its high anti-*Pseudomonal* activity also in physiologically relevant conditions [46,51] makes this short-sized AMP a particularly attractive candidate wound healing promoter, especially in the management of chronic, often *Pseudomonas*-super-infected, human skin ulcers [52,100].
